# The Association between Percentage of Mean Arterial Pressure and Long-Term Mortality in Acute Myocardial Infarction Patients: An Observational Cohort Study

**DOI:** 10.7150/ijms.95430

**Published:** 2024-08-12

**Authors:** Yi-Hsueh Liu, Wei-Chung Tsai, Nai-Yu Chi, Ching-Tang Chang, Wen-Hsien Lee, Chun-Yuan Chu, Tsung-Hsien Lin, Sheng-Hsiung Sheu, Ho-Ming Su, Po-Chao Hsu

**Affiliations:** 1 Division of Cardiology, Department of Internal Medicine, Kaohsiung Medical University Hospital, Kaohsiung, Taiwan.; 2 Department of Internal Medicine, Kaohsiung Municipal Siaogang Hospital, Kaohsiung, Taiwan.; 3 Faculty of Medicine, College of Medicine, Kaohsiung Medical University, Kaohsiung, Taiwan.

**Keywords:** acute myocardial infarction, cardiovascular, mortality, percentage of mean arterial pressure, ankle-brachial index

## Abstract

**Background:** Acute myocardial infarction (AMI) is a critical cardiovascular disease with high morbidity and mortality. Identifying practical parameters for predicting long-term mortality is crucial in this patient group. The percentage of mean arterial pressure (%MAP) is a useful parameter used to assess peripheral artery disease. It can be easily calculated from ankle pulse volume recording. Previous studies have shown that %MAP is a useful predictor of all-cause mortality in specific populations, but its relationship with mortality in AMI patients is unclear.

**Methods:** In this observational cohort study, 191 AMI patients were enrolled between November 2003 and September 2004. Ankle-brachial index (ABI) and %MAP were measured using an ABI-form device. All-cause and cardiovascular mortality data were collected from a national registry until December 2018. Cox proportional hazards model and Kaplan-Meier survival plot were used to analyze the association between %MAP and long-term mortality in AMI patients.

**Results:** The median follow-up to mortality was 65 months. There were 130 overall and 36 cardiovascular deaths. High %MAP was associated with increased overall mortality after multivariable analysis (HR = 1.062; 95% CI: 1.017-1.109; *p* =0.006). However, high % MAP was only associated with cardiovascular mortality in the univariable analysis but became insignificant after the multivariable analysis.

**Conclusions:** In conclusion, this study is the first to evaluate the usefulness of %MAP in predicting long-term mortality in AMI patients. Our study shows that %MAP might be an independent predictor of long-term overall mortality in AMI patients and has better predictive power than ABI.

## Introduction

Acute myocardial infarction (AMI) is a major cause of mortality worldwide and can result in significant health-related burden 1. In Taiwan, cardiovascular disease (CVD) has been the second leading cause of mortality since 2010, and AMI is the most severe form of CVD 2. Patients in Taiwan with ST-segment elevation myocardial infarction (STEMI) and Non-ST-segment elevation myocardial infarction (NSTEMI) had a 1-year mortality rate of 6.1% and 10.1%, respectively 3. Peripheral artery disease (PAD) is a coexisting condition that increases cardiovascular risk and mortality in patients with AMI 4, 5. However, it is under-diagnosed as 40%-50% of patients with PAD are asymptomatic 6, 7.

The ankle-brachial index (ABI), the ratio of ankle-to-brachial systolic blood pressure, is a commonly used non-invasive diagnostic tool for PAD 8, 9. The current guidelines recommend that resting ABI results be classified as abnormal (ABI ≤0.90), borderline (ABI 0.91-0.99), normal (1.00-1.40), or noncompressible (ABI >1.40) 8, 9. Moreover, the ABI has been shown to be a useful predictor for risk of cardiovascular disease events and all-cause mortality 10. However, the sensitivity of ABI to detect PAD is limited in patients with diabetes or chronic kidney disease (CKD) because calcification of the arteries can result in vascular inability to be compressed. 11-13. Ultimately, finding a reliable and user-friendly tool is essential to aid in the early detection of subclinical PAD and prevent further cardiovascular events.

The percentage of mean arterial pressure (%MAP) is a convenient index for screening for PAD. It can be calculated from a pulse volume recording at the ankle and automatically reported by the ABI-measuring machine. The %MAP is calculated as the percentage of the area under the pulse waveform relative to the area of the rectangle that encloses the pulse waveform 14-16. The %MAP indicates a flattened arterial wave and can serve as a diagnostic criterion for PAD in patients with falsely elevated ABI values due to noncompressible vessels 16, 17. Combining the ABI and the %MAP can help improve the diagnostic sensitivity of PAD 18, 19. In a previous study primarily composed of individuals with diabetes and the elderly, a higher %MAP was found to correlate with increased mortality risk among participants with normal range ABI values 14. The main value of %MAP may be that it is not affected by noncompressible arteries. Even within the normal range of ABI values, patients with a higher %MAP may better reflect the actual characteristics of arterial occlusion and arteriosclerosis. In addition, %MAP is also a useful predictor of all-cause mortality in specific populations, including diabetes and hemodialysis patients 20, 21. However, the relationship between %MAP and mortality has not yet been assessed in participants with AMI. Therefore, the present study aims to determine whether the %MAP predicts long-term cardiovascular and overall mortality in AMI patients.

## Materials and Methods

### Study population and design

This observational cohort study enrolled patients diagnosed with AMI between November 2003 and September 2004 in our cardiac care unit. Both STEMI and NSTEMI patients were included in the study, with the minimum age requirement being 20 years. The authors had access to information that could identify individual participants during or after data collection. Patients were excluded from the study if they had unstable hemodynamic status, atrial fibrillation, limb amputation, or missing data on four-limb blood pressures, ABI, or %MAP. Finally, the study included a total of 191 AMI patients. All cause and cardiovascular mortality data were obtained from the Collaboration Center of Health Information Application, Ministry of Health and Welfare, Executive Yuan, Taiwan, and were collected up to December 2018. The medical information and data were accessed for research purposes on February 25, 2020.

### Ethics statement

The research protocol was approved by the institutional review board (IRB) committee of Kaohsiung Medical University (KMUHIRB-E(I)-20190258). All patients provided written informed consent during the first day of admission, and all clinical investigations adhered to the principles outlined in the Declaration of Helsinki.

### Assessment of ABI, %MAP, and four limb blood pressures by ABI-form device

The ABI, %MAP and blood pressures in all four limbs were measured using an ABI-form device (VP1000; Colin Co. Ltd., Komaki, Japan) that employs an oscillometric method to measure blood pressure in both arms and ankles simultaneously after resting in a supine position for at least 5 minutes 22, 23. Cuffs were placed tightly around the upper arms and ankles for occlusion and monitoring purposes without accessing blood. The ABI of each leg was determined by dividing the ankle systolic blood pressure by the higher brachial systolic blood pressure, and the lower of the two ABI values was used for data analysis. The %MAP was automatically determined from the pulse volume recordings by using the following formula 16:


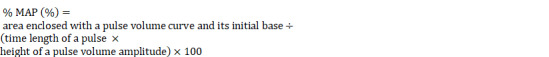
)

The higher %MAP between both legs was selected for analysis. The ABI-form device measurement was performed once within 24 hours of admission to the cardiac care unit for each patient.

### Collection of demographic and medical data

Demographic and medical information, such as age, gender, and comorbidities like dyslipidemia, diabetes, and hypertension, were obtained from the patients' medical records.

### Statistical analysis

Statistical analyses were conducted using SPSS 22.0 software (SPSS, Chicago, IL, USA). The data were presented as mean ± standard deviation, percentage, or median (25th-75th percentile) for the follow-up period. The independent samples t-test and Chi-square test were used to compare continuous and categorical variables between groups, respectively. In our study, we performed both univariable and multivariable Cox proportional hazards regression analyses to determine the predictors of overall and cardiovascular mortality. In the univariable analysis, each variable was analyzed independently to assess its association with the outcomes. Variables that were found to be significant in the univariable analysis (p < 0.05) were then included in the multivariable analysis to control for potential confounders and assess the independent effect of each variable on mortality. Kaplan-Meier survival plot was calculated from baseline to time of mortality events. All tests were 2-sided and P value less than 0.05 was considered statistically significant.

### Adjustment for confounders

In our multivariable Cox proportional hazards regression models, we adjusted for several potential confounders, including age, gender, hypertension, heart rate, BMI, LVEF, ABI, and %MAP. These factors were selected based on their known associations with cardiovascular outcomes and mortality in the literature. The theoretical basis for these adjustments is to control for variables that could influence both the exposure (%MAP) and the outcomes (overall and cardiovascular mortality), thus providing a clearer understanding of the independent effect of %MAP. In addition, only significant variables in the univariate analysis were included in the multivariate analysis.

## Results

Of the 191 subjects (139 males and 52 females) included in this study, the mean age of 65.8 ± 13.5 years for our study population is consistent with the reported average age for patients with AMI in Taiwan 24. Among them, 36 patients had STEMI while 155 had NSTEMI. The median follow-up period for mortality was 65 months, with a range of 6 to 174 months (25th-75th percentile). There were 130 and 36 patients documented as all cause and cardiovascular mortality, respectively.

**Table 1** shows a comparison of the clinical characteristics between survivors and non-survivors. Compared to the survivors, the non-survivors were older, had a higher prevalence of female gender, higher prevalence rates of hypertension, higher heart rate, higher body mass index (BMI), lower left ventricular ejection fraction (LVEF), lower ABI (0.91 ± 0.22 versus 1.03 ± 0.11, p < 0.001), and higher %MAP (44.1 ± 7.8 versus 36.6 ± 5.0, p < 0.001).

**Table 2** shows the predictors of overall mortality using Cox proportional hazards model in the univariable and multivariable analysis. In our univariable analysis, older age, female gender, presence of hypertension, increased heart rate, lower body mass index, lower LVEF, lower ABI, and higher %MAP were significant predictors of overall mortality. After multivariable analysis adjusted for age, gender, hypertension, heart rate, BMI, LVEF, ABI, and %MAP (significant variables in univariable analysis), only age (hazard ratio [HR] = 1.047; 95% confidence interval [CI]: 1.022-1.073; p < 0.001), LVEF (HR = 0.981; 95% CI: 0.965-0.998; p = 0.025), and %MAP (HR = 1.062; 95% CI: 1.017-1.109; p = 0.006) were significantly associated with overall mortality. Lower ABI became insignificant after multivariable analysis (*p* = 0.712).

**Table 3** shows the predictors of cardiovascular mortality using Cox proportional hazards model in the univariable and multivariable analysis. In our univariable analysis, older age, female gender, higher percentage of hypertension, increased heart rate, lower ABI, and higher %MAP were significant predictors of cardiovascular mortality. After multivariable analysis adjusted for age, gender, hypertension, heart rate, ABI and %MAP (significant variable in univariable analysis), only age (HR = 1.038; 95% CI: 1.001-1.076; *p* =0.043) was significantly associated with cardiovascular mortality. Lower ABI (*p* = 0.652).and higher %MAP (*p* =0.990) became insignificant after multivariable analysis.

**Figure 1:**
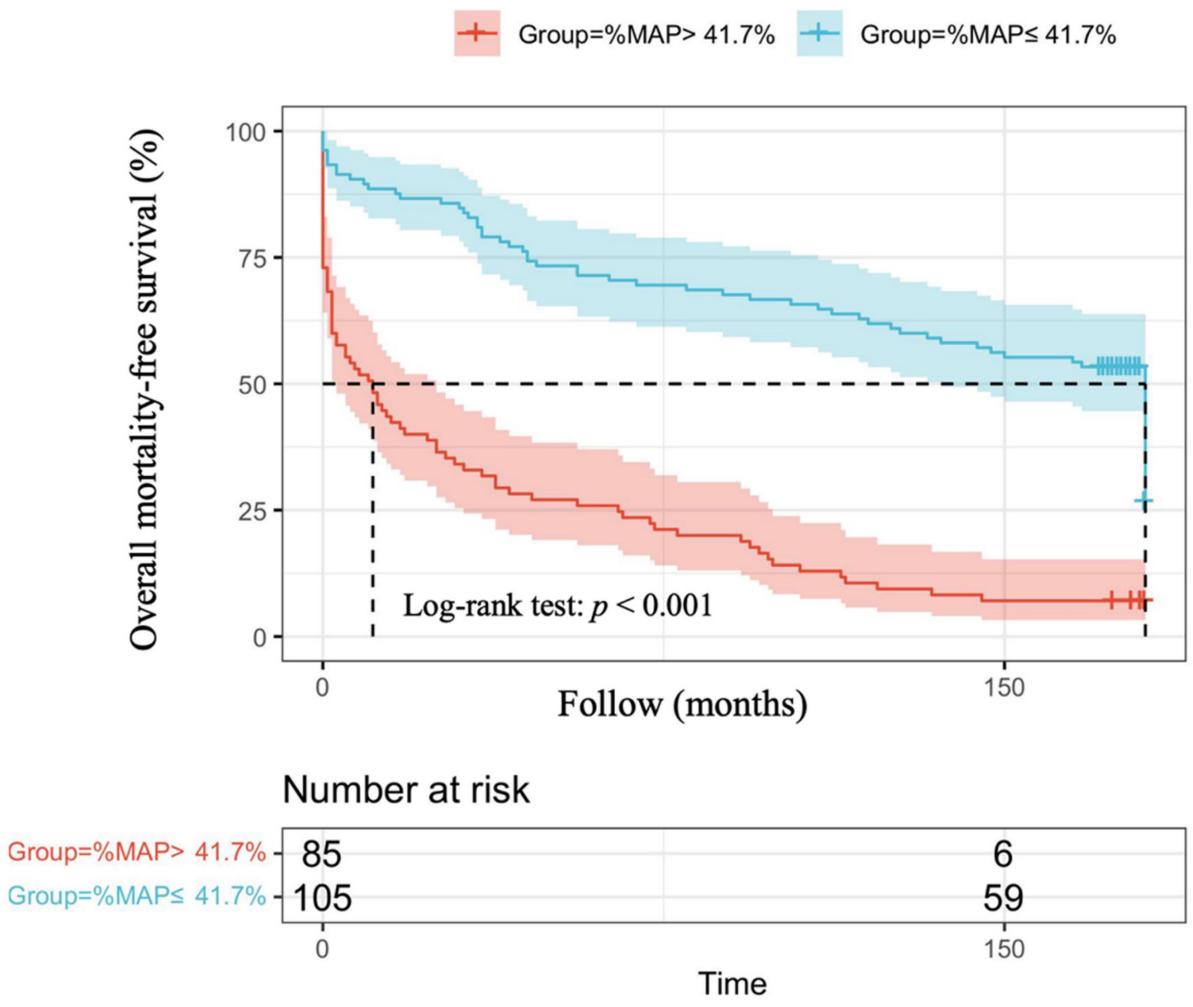
Adjusted Kaplan-Meier curves of %MAP above the median versus below the median for overall mortality-free survival (log-rank p<0.001).

**Figure 2:**
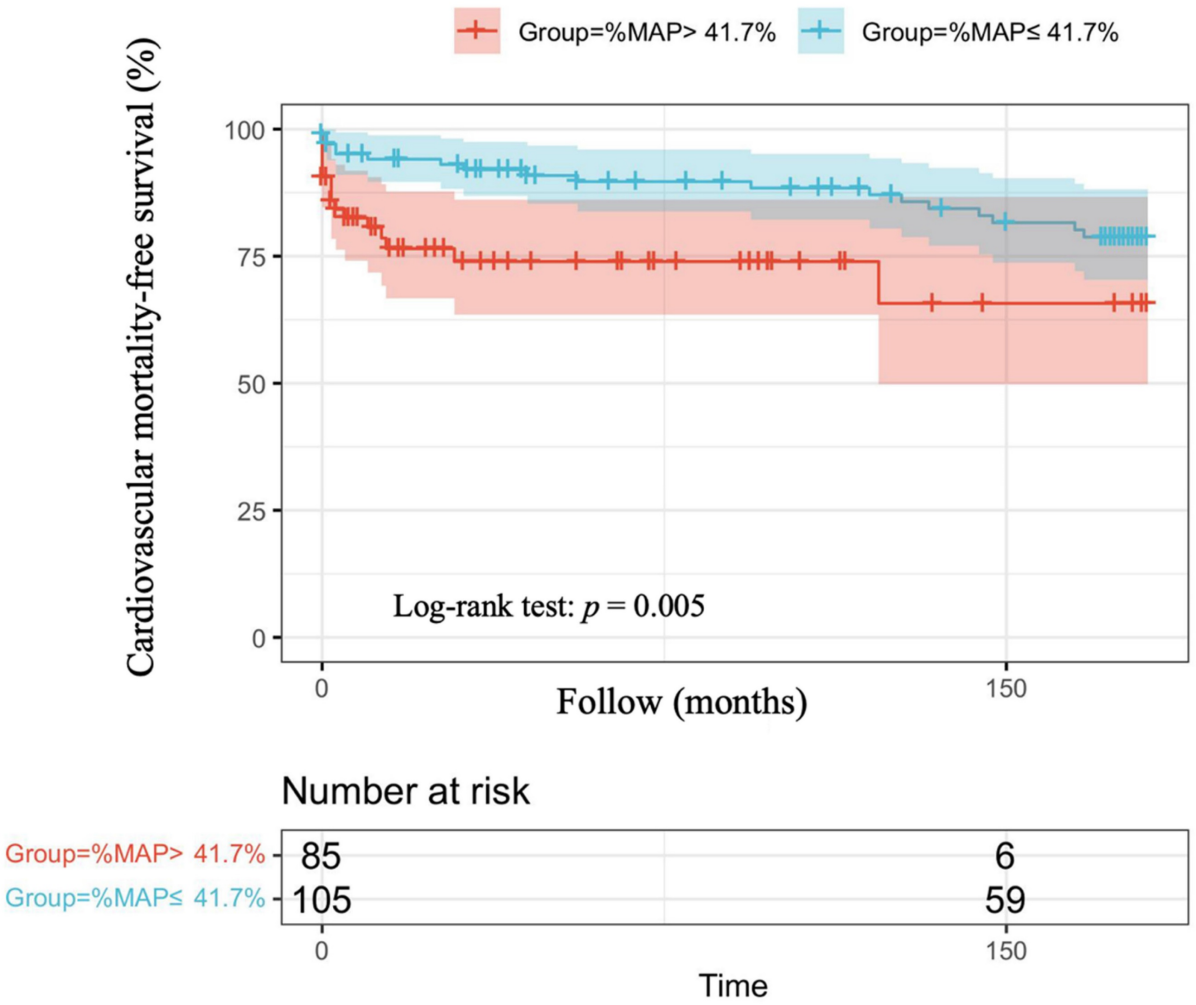
Adjusted Kaplan-Meier curves of %MAP above the median versus below the median for cardiovascular mortality-free survival (log-rank p=0.005).

The optimal cut-off vales of %MAP and for the prediction of overall mortality and cardiovascular mortality have not been established in patients with AMI. Therefore, we used the mean value of %MAP as the cut-off, resulting in 85 patients with %MAP greater than the mean value (%MAP>41.7%) and 105 patients with %MAP less than or equal to the mean value (%MAP≤41.7%). Figure 1 illustrates the adjusted Kaplan-Meier curves of %MAP above the median versus below the median for overall mortality-free survival (log-rank p < 0.001). Figure 2 shows the adjusted Kaplan-Meier curves of %MAP above the median versus below the median for cardiovascular mortality-free survival (log-rank p = 0.005).

## Discussion

The aim of our study was to assess the predictive value of %MAP for overall and cardiovascular mortality in AMI patients. Our study yielded several key findings. First, higher %MAP was found to be associated with an increased risk of overall mortality in both univariable and multivariable analysis. Second, in univariable analysis, higher %MAP was also associated with increased cardiovascular mortality, although this association became insignificant in multivariable analysis. Third, while ABI was found to be associated with increased total and cardiovascular mortality in univariable analysis, it lost its significance when multivariable analyses were added to %MAP. Finally, %MAP demonstrated better predictive value than conventional parameters, such as ABI and LVEF, for overall mortality prediction in AMI patients.

Over the past decade, the prognosis of patients with AMI has improved due to the adoption of guideline-recommended treatment methods such as early vascular revascularization, anti-thrombotic therapy, and other secondary prevention measures 25-27. Most data focus on events and predictors within the first year after AMI 28-30, and there is still little understanding of which risk factors can influence long-term cardiovascular events and all-cause mortality after the onset of AMI 31, 32. There are many traditional risk factors that have been found to be associated with short-term and long-term cardiovascular prognosis, such as age, gender, renal impairment, diabetes, heart rate, BMI and LVEF 28, 29, 31-33. Our study also found that these traditional factors affect long-term all-cause mortality, in line with previous studies.

Patients with AMI who also have clinical PAD are at a higher risk of mortality during their index hospitalization 34 as well as during long-term follow-up 5, 35. Previous studies reported that both subclinical and clinical PAD are associated with a poor prognosis in patients with AMI, indicating that routine ABI testing could be prognostically significant regardless of PAD symptoms 4, 36. The association between ABI and mortality has a U-shaped curve rather than a linear correlation 37, 38. In patients with AMI, those with low ABI have a higher mortality rate 4, 5. However, the predictive ability of ABI varies among different comorbid populations. Low ABI was linked to mortality in both individuals with and without diabetes in the REACH registry, while the association with high ABI was only apparent in patients with diabetes 39. However, in certain patient populations, such as those with diabetes mellitus or CKD, the ABI may be false elevated due to vascular calcification 11-13, 40. This could affect the diagnostic accuracy and consequently the accuracy of the ABI as a prognostic factor. In our study, low ABI was found to be associated with both overall and cardiovascular mortality in univariable analysis, but this association was not statistically significant in the multivariable analysis.

The ABI-form (VP 1000; Colin Co Ltd, Komaki, Japan) is a clinical device that utilizes an automated oscillometric method to measure blood pressures in all four limbs and record pulse waves simultaneously and automatically 16. This device simultaneously recorded four limbs blood pressure, ABI, electrocardiogram, phonocardiogram and % MAP. Pulse volume recording is a viable alternative tool for diagnosing PAD with calcified vessels 41, and its main value may be that it is unaffected by the presence of incompressible arteries 17. This is achieved by using pneumatic cuffs to inject a standard volume of air and occlude the venous circulation to solely detect volume changes related to arterial circulation, as translated by the transducer into a pulsatile pressure waveform 42. Li et al. were the first to apply ankle %MAP to predict mortality and found that a high %MAP obtained from pulse volume recording was a strong predictor of all-cause mortality in participants with an ABI value in the range of 0.9-1.3, after adjusting for various confounding factors 14. Lee et al. reported that increased %MAP and %MAP > 50% were significant predictors of total mortality, while %MAP > 50% was a predictor of cardiovascular mortality. These findings suggest that assessing %MAP from pulse volume recording at the ankle could be a useful tool for identifying high-risk hemodialysis patients for poor prognosis 21. For type 2 diabetes patient, Li et a. also found that the %MAP in combination with the ABI can improve the prediction of mortality. The study retrospectively collected data from 5569 patients and found that the combination of ABI and %MAP was more effective than ABI alone in predicting mortality. In multivariate analysis, the highest risk of mortality was seen in patients with ABI ≤ 0.90 and %MAP > 45%. Therefore, using %MAP in conjunction with ABI could improve the prediction of all-cause mortality in patients with type 2 diabetes 20.

In our present study, high %MAP was found to be significantly associated with long-term overall mortality in both univariable and multivariable analyses among patients with AMI. Although a significant difference was observed in the association between high %MAP and long-term cardiovascular mortality in patients with AMI in univariable analyses, such a difference was not seen in multivariable analyses after adjusting for various confounding factors. In our analysis of the Kaplan Meier survival curve, we also found that AMI patients with a %MAP greater than the mean value (41.7 %) had a higher overall mortality. Therefore, in our study, we found that among patients with AMI, %MAP was a potential parameter that could be used as a predictor of overall mortality.

### Study limitations

There are several limitations to this study. First, the study generality was limited because the study included only patients from the intensive care unit of single medical center in southern Taiwan. Second, we did not assess the association of peripheral arterial occlusive disease with %MAP in the image study. Third, the sample size of our study was not very large, but the follow-up period was long, up to 181 months. Fourth, our study was missing many cardiovascular risk factors from the characteristics at baseline, such as continuous blood pressure, LDL, and glucose values at the baseline followed by smoking history and drug history. Due to incomplete data, we did not adjust for many biochemical markers and cardiovascular risk factors in our univariate and multivariate analyses. This is because at our hospital, detailed medical records of patients are not available if they do not return to our hospital for more than 10 years. Fifth, we have solely analyzed outcomes such as cardiovascular mortality and overall mortality; however, we lack relevant data on cardiovascular morbidity, major adverse cardiovascular events, and stroke to thoroughly evaluate the outcomes. Sixth, in this study, men and women were analyzed together due to the limited sample size. We acknowledge that gender differences in cardiovascular risk factors could influence the outcomes. Future studies with larger sample sizes will include sex-stratified analyses or interaction tests to explore gender-specific effects. Finally, we conducted a post hoc power analysis to determine the adequacy of our sample size. The power analysis indicated that our study had sufficient power to detect significant associations between %MAP and overall mortality but might be underpowered for some subgroup analyses, such as cardiovascular mortality and sex-stratified analyses. This limitation underscores the need for larger sample sizes in future studies to confirm our findings and explore additional outcomes.

## Conclusions

Our study is the first study to evaluate the usefulness of %MAP in AMI patients for prediction of long-term overall and cardiovascular mortality. Our study showed %MAP might be used as a potential parameter to predict long-term overall mortality among AMI patients. In addition, it also has a better additive predictive value for long-term mortality than ABI and LVEF in AMI patients. Thus, utilizing %MAP as a simple screening tool in AMI patients may assist clinicians in identifying individuals at high risk of increased mortality.

## Figures and Tables

**Table 1:** Comparison of baseline characteristics between Survivors and Non-survivors

Characteristics	Survivors (*n* =61)	Non-survivors(*n* =130)	*p* value	All patients(*n*= 191)
Age (years)	55.3 ± 11.6	70.7 ± 11.5	< 0.001	65.8 ± 13.5
Male gender (%)	83.6	67.7	0.013	72.8
Hypertension (%)	27.9	47.7	0.007	41.4
Diabetes mellitus (%)	24.6	28.5	0.578	27.2
Dyslipidemia (%)	69.2	64.7	0.683	66.0
STEMI (%)	21.3	17.7	0.553	18.9
NSTEMI (%)	78.7	82.3	0.553	71.1
Heart rate (beat/min)	73.8 ± 14.4	81.2 ± 17.8	0.003	78.8 ± 17.1
Body mass index (kg/m^2^)	25.6 ± 3.2	23.6 ± 3.9	0.001	24.2 ± 3.8
LVEF (%)	63.7 ± 12.5	55.7 ± 16.2	0.002	58.9 ± 15.3
Ankle brachial index	1.03 ± 0.11	0.91 ± 0.22	< 0.001	0.95 ± 0.20
%MAP	36.6 ± 5.0	44.1 ± 7.8	< 0.001	41.7 ± 7.8

Abbreviations: STEMI, ST-segment elevation myocardial infarction; NSTEMI, non-ST elevation myocardial infarction; LVEF, left ventricular ejection fraction; %MAP, percentage of mean arterial pressure

**Table 2:** Predictors of overall mortality using Cox proportional hazards model by univariable and multivariable analysis

Parameter	Univariable analysis		Multivariable analysis	
	HR (95% CI)	*P*	HR (95% CI)	*P*
Age (Per 1 year)	1.074 (1.057-1.091)	<0.001	1.047 (1.022-1.073)	<0.001
Gender (Male vs. Female)	0.602 (0.416-0.872)	0.007	1.113 (0.618-2.004)	0.721
Hypertension (Yes vs. No)	1.505 (1.066-2.125)	0.020	0.950 (0.580-1.558)	0.840
Diabetes mellitus (Yes vs. No)	1.143 (0.778-1.680)	0.496	-	-
Dyslipidemia (Yes vs. No)	0.773 (0.469-1.274)	0.312	-	-
STEMI (Yes vs. No)	0.881 (0.561-1.384)	0.583	-	-
Heart rate (Per beat/min)	1.018 (1.008-1.028)	<0.001	1.011 (0.996-1.026)	0.162
Body mass index (Per 1kg/m^2^)	0.897 (0.853-0.944)	<0.001	0.966 (0.904-1.031)	0.296
LVEF (Per 1%)	0.983 (0.969-0.997)	0.016	0.981 (0.965-0.998)	0.025
Ankle brachial index (Per 1SD)	0.073 (0.031-0.170)	<0.001	1.382 (0.247-7.719)	0.712
%MAP (Per 1SD)	1.087 (1.064-1.110)	<0.001	1.062 (1.017-1.109)	0.006

HR: hazard ratio; CI: confidence interval; SD: standard deviation; other abbreviations as in Table 1.

**Table 3:** Predictors of cardiovascular mortality using Cox proportional hazards model by univariable and multivariable analysis

Parameter	Univariable analysis		Multivariable analysis	
	HR (95% CI)	*P*	HR (95% CI)	*P*
Age (Per 1 year)	1.051 (1.021-1.082)	0.001	1.038 (1.001-1.076))	0.043
Gender (Male vs. Female)	0.462 (0.236-0.908)	0.025	0.753 (0.342-1.657)	0.481
Hypertension (Yes vs. No)	2.592 (1.323-5.077)	0.005	1.717 (0.837-3.523)	0.140
Diabetes mellitus (Yes vs. No)	1.150 (0.554-2.387)	0.707	-	-
Dyslipidemia (Yes vs. No)	0.867 (0.349-2.151)	0.758	-	-
STEMI (Yes vs. No)	1.005 (0.440-2.297)	0.990	-	-
Heart rate (Per 1beat/min)	1.019 (1.001-1.038)	0.040	1.016 (0.996 - 1.036)	0.124
Body mass index (Per 1kg/m^2^)	0.955 (0.869-1.049)	0.337	-	-
LVEF (Per 1%)	0.978 (0.950-1.006)	0.126	-	-
Ankle brachial index (Per 1SD)	0.160 (0.030-0.846)	0.031	0.599 (0.065-5.548)	0.652
%MAP (Per 1SD)	1.051 (1.007-1.097)	0.023	1.000 (0.934-1.072)	0.990

HR: hazard ratio; CI: confidence interval; SD: standard deviation; other abbreviations as in Table 1.

## Abbreviations

AMI: acute myocardial infarction
CVD: cardiovascular disease
STEMI: ST-segment elevation myocardial infarction
NSTEMI: Non-ST-segment elevation myocardial infarction
PAD: Peripheral artery disease
ABI: ankle-brachial index
CKD: chronic kidney disease
%MAP: percentage of mean arterial pressure
BMI: body mass index
LVEF: left ventricular ejection fraction
